# CRISPR/Cas9-edited *ROS1* + non-small cell lung cancer cell lines highlight differential drug sensitivity in 2D vs 3D cultures while reflecting established resistance profiles

**DOI:** 10.1186/s12967-024-04988-0

**Published:** 2024-03-03

**Authors:** Marc Terrones, Christophe Deben, Felicia Rodrigues-Fortes, Anne Schepers, Ken Op de Beeck, Guy Van Camp, Geert Vandeweyer

**Affiliations:** 1 Center of Medical Genetics, University of Antwerp and Antwerp University Hospital, Edegem, Belgium; 2 Center for Oncological Research, University of Antwerp and Antwerp University Hospital, Wilrijk, Belgium

**Keywords:** *ROS1* + Non-small cell lung cancer, Tyrosine kinase inhibitors, CRISPR/Cas9, Drug resistance, 3D cell culture, 2D cell culture

## Abstract

**Introduction:**

The study of resistance-causing mutations in oncogene-driven tumors is fundamental to guide clinical decisions. Several point mutations affecting the ROS1 kinase domain have been identified in the clinical setting, but their impact requires further exploration, particularly in improved pre-clinical models. Given the scarcity of solid pre-clinical models to approach rare cancer subtypes like *ROS1* + NSCLC, CRISPR/Cas9 technology allows the introduction of mutations in patient-derived cell lines for which resistant variants are difficult to obtain due to the low prevalence of cases within the clinical setting.

**Methods:**

In the *SLC34A2-ROS1* rearranged NSCLC cell line HCC78, we knocked-in through CRISPR/Cas9 technology three ROS1 drug resistance-causing mutations: G2032R, L2026M and S1986Y. Such variants are located in different functional regions of the ROS1 kinase domain, thus conferring TKI resistance through distinct mechanisms. We then performed pharmacological assays in 2D and 3D to assess the cellular response of the mutant lines to crizotinib, entrectinib, lorlatinib, repotrectinib and ceritinib. In addition, immunoblotting assays were performed in 2D-treated cell lines to determine ROS1 phosphorylation and MAP kinase pathway activity. The area over the curve (AOC) defined by the normalized growth rate (NGR_fit) dose–response curves was the variable used to quantify the cellular response towards TKIs.

**Results:**

Spheroids derived from ROS1^G2032R^ cells were significantly more resistant to repotrectinib (AOC fold change = − 7.33), lorlatinib (AOC fold change = − 6.17), ceritinib (AOC fold change = − 2.8) and entrectinib (AOC fold change = − 2.02) than wild type cells. The same cells cultured as a monolayer reflected the inefficacy of crizotinib (AOC fold change = − 2.35), entrectinib (AOC fold change = − 2.44) and ceritinib (AOC fold change = − 2.12) in targeting the ROS1 G2032R mutation. ROS1^L2026M^ cells showed also remarkable resistance both in monolayer and spheroid culture compared to wild type cells, particularly against repotrectinib (spheroid AOC fold change = − 2.19) and entrectinib (spheroid AOC fold change = − 1.98). ROS1^S1986Y^ cells were resistant only towards crizotinib in 2D (AOC fold change = − 1.86). Overall, spheroids showed an increased TKI sensitivity compared to 2D cultures, where the impact of each mutation that confers TKI resistance could be clearly distinguished. Western blotting assays qualitatively reflected the patterns of response towards TKI observed in 2D culture through the levels of phosphorylated-ROS1. However, we observed a dose–response increase of phosphorylated-Erk1/2, suggesting the involvement of the MAPK pathway in the mediation of apoptosis in HCC78 cells.

**Conclusion:**

In this study we knock-in for the first time in a *ROS1* + patient-derived cell line, three different known resistance-causing mutations using CRISPR/Cas9 in the endogenous translocated *ROS1* alleles. Pharmacological assays performed in 2D and 3D cell culture revealed that spheroids are more sensitive to TKIs than cells cultured as a monolayer. This direct comparison between two culture systems could be done thanks to the implementation of normalized growth rates (NGR) to uniformly quantify drug response between 2D and 3D cell culture. Overall, this study presents the added value of using spheroids and positions lorlatinib and repotrectinib as the most effective TKIs against the studied ROS1 resistance point mutations.

**Supplementary Information:**

The online version contains supplementary material available at 10.1186/s12967-024-04988-0.

## Introduction

*ROS1* + non-small cell lung cancers (NSCLC) account for 2% of newly diagnosed cases, arising as a result of a chromosomal translocation that leads to the formation of an oncogenic fusion protein [1]. A remarkable diversity of ROS1 fusions has been reported in NSCLC patients, where the *ROS1* fusion gene partner seems responsible for defining the subcellular localization of the resulting protein. In consequence, the partner gene will determine the downstream signaling pathway to be activated [2]. The ROS1 tyrosine kinase domain is conserved in all of them which drives cell growth, proliferation and migration via the interaction with the MAP kinase, JAK/STAT and mTOR/AKT pathways. These classical cell signaling cascades are responsible for enhancing cell growth, proliferation and increased cell survival [3]. At a therapeutical level, *ROS1* + NSCLC can be drugged in a targeted manner through tyrosine kinase inhibitors (TKIs). These are small molecules that selectively prevent the phosphorylation by the ROS1 kinase of its downstream interactor proteins like SH2. The blocking of the oncogenic signaling eventually leads to tumor shrinkage [4, 5]. There are currently 4 TKIs FDA- approved (crizotinib, entrectinib, ceritinib and repotrectinib) to treat *ROS1* + NSCLC. At a European level only crizotinib,and entrectinib have been authorized, whilst repotrectinib recently underwent marketing authorization application [6–9]. However, other TKIs targeting oncogenic kinases that share a certain degree of homology with ROS1 like lorlatinib (use to treat *ALK* + NSCLC) or next-generation TKIs like NVL-520 have been proven to be effective in *ROS1* + NSCLC patients [10, 11]. In consequence, the available TKIs remain limited and their regulatory status is internationally heterogeneous.

Despite an outstanding initial response to TKIs, subclonal neoplastic cell populations can acquire mutations as a result of the selective pressure partially mediated by the TKIs, leading to treatment resistance. Depending on the nature of the mutations, they are classified as extrinsic or intrinsic resistance mechanisms. The first type involves alterations in genes other than *ROS1*; like *MYC* or *MET* amplification, overactivation of the EGFR signaling pathway or events like epithelial-to-mesenchymal transition (EMT) [1]. The second class of resistance mechanisms, also known as intrinsic, impact the ROS1 kinase domain of the fusion. Amino acid substitutions like G2032R, F2004V or L1951R lead to conformational changes of different regions within the ROS1 kinase domain that modulate its interactions with TKIs [12–14]. Among the most prevalent mutations reported in *ROS1* + NSCLC patients who relapse upon treatment, the solvent front mutations G2032R (around 40% of cases) and D2033N (6%), together with the gatekeeper mutation L2026M (6%) can be found. They confer steric interference with drug binding, being located in key functional regions of the ROS1 kinase domain [15]. Interestingly, new functional implications of the G2032R and L2026M variants have been recently demonstrated concerning *CD74-ROS1*-rearranged NSCLC. Gou et al. showed that the G2032R variant upregulates the expression of *TWIST1,* a gene that encodes a transcription factor responsible for orchestrating epithelial-to-mesenchymal transition (EMT) [16]. Similarly, Xu et al. reported that the L2026M variant contributes towards TKI resistance by stimulating autophagy, mediated by the MEK/ERK pathway [17]. With a comparable prevalence, substitutions like S1986Y/F (6%), described as a kinase over-activating mutation, L2000V or L2086F have been also reported. However, being located outside the active site of the kinase, their impact in TKI activity remains poorly understood [10, 12, 18]. Available studies indicates that each mutation has a different impact on the interaction between the ROS1 kinase and TKIs. Thus, a thorough mutation-based approach is required to define their contribution in TKI resistance.

Several pre-clinical models have been developed to characterize molecular changes in neoplastic cells driven by mutations acquired within the kinase domain. The pro-B-cell, murine-derived Ba/F3 cell line has been widely used to express exogenous mutant isoforms of *ROS1* oncogenic translocations [19]. In addition, the A549 cell line, a NSCLC line expressing ROS1 wild type and KRAS G12S has also been transformed using the same approach as with Ba/F3 cells [16]. Nevertheless, these lines offer a limited representation of *ROS1* + NSCLC for several reasons. Firstly, the expression levels of the exogenous ROS1 fusion are remarkably high since they are regulated by a CMV promoter; which differs from the regulatory elements of the ROS1 fusion gene partners. Secondly, the aforementioned models also neglect factors present in the genetic background of patients, key to study drug response. For instance, Ba/F3 cells have a murine origin and they belong to the hematopoietic lineage; poorly recapitulating the molecular landscape of alterations in *ROS1* + NSCLC. In contrast, HCC78 was the first available patient-derived *ROS1* + cell line. It has been used in several studies and it is genetically well characterized [20–23]. Importantly, HCC78 spheroids have been used to assess the response to TKIs in *ROS1* wild type cells [24]. Hence, HCC78 is a prime candidate to to reproduce in vitro the resistant phenotypes observed in patients presenting the three selected variants. By studying the variants in both 2D and 3D cell cultures, potentially relevant changes in response rates can be identified related to the more natural growth pattern. Within the field of lung cancer, the CRISPR/Cas9 methodology has been implemented to model the EGFR T790M variant; which leads to gefitinib resistance [25]. Nonetheless, no CRISPR/Cas9 generated ROS1 resistant models have been studied in both 2D and 3D.

Besides replacing the traditional cell transformation with CRISPR/Cas9-mediated gene editing, an additional innovation to be implemented in the field of drug screenings involved the assessment of the TKI activity in cell cultures. The majority of the cell viability assays are performed through measurements of the metabolic activity of cells, as a surrogate of the proportion of alive cells (e.g. the colorimetric assay MTT). However, imaging-based approaches such as the normalized growth rate (NGR) and the area over the curve (AOC) defined by the NGR emerged as novel metrics. They have been proven to be more accurate since they allow the differential detection of cytotoxic and cytostatic cellular responses; as opposed to conventional half-maximal inhibitory concentration (IC50) [26, 27]. In order to complement the observations in the drug assays, the immunoblotting of the phosphorylated ROS1 (p-ROS1) and Erk1/2 (p-Erk1/2) is incorporated to accurately profile the inhibitory activity of TKIs.

In conclusion, this study aims to refine the pre-clinical *ROS1* + NSCLC models by knocking-in resistance mutations G2032R, L2026M and S1986Y via CRISPR/Cas9 in the *ROS1* + patient-derived HCC78 cell line, followed by functional characterization and pharmacological assays in 2D and 3D cultures.

## Methods

### Cell lines

HCC78 cells were obtained from the German Collection of Microorganisms and Cell Cultures GmbH (DSMZ). They were cultured in RPMI 1640 containing L-glutamine, HEPES and supplemented with 10% FBS (v/v). Cells were kept in a humidified incubator at 37 degrees, identity-verified via STR analysis using the GenePrint® 10 System kit (Promega #B9510) and regularly tested for *Mycoplasma* infection using the LookOut® Mycoplasma PCR detection kit (Sigma Aldrich #MP0035). The last passage numbers of each cell line used in the drug screenings and immunoblotting are: HCC78 ROS1 wild type: p24, ROS1 G2032R: p29, ROS1 L2026M: p25 and ROS1 S1986Y: p30.

### CRISPR/Cas9-mediated gene editing

Per knock-in, 5.10^6^ cells were electroporated using the SF Nucleofector kit with the CM-130 program using 20 pmol of ribonucleoprotein in a gRNA:Cas9 ratio of 1:1.2. The Cell Line Nucleofection Optimization Kit (Lonza) was applied to select an optimal nucleofection program, but resulted in low nucleofection efficiencies (< 60% GFP + cells) (Additional file 1: Fig. S1a). Quantification was done using CytoFLEX flow cytometer (Beckman Coulter) upon cell resuspension in FACS buffer and staining with propidium iodide. 10,000 events were recorded per sample and data was analyzed with CytExpert (Beckman Coulter) and FlowJo (BD Biosciences) softwares. The CM-130 program was eventually selected since it was reported in a study using A549 cells. The authors defined it as the most effective program based on additional experiments performed in other lung adenocarcinoma cell lines [28]. gRNAs were designed using CHOPCHOP (https://chopchop.cbu.uib.no/) and IDT (https://eu.idtdna.com/site/order/designtool/index/CRISPR_CUSTOM). The gRNAs chosen for each knock-in were independently designed by two alternative tools to ensure their functionality. In addition, 25 µM of ssODN (DNA donor template containing the variant of interest designed using IDT’s online tool as well) and 25 µM of IDT electroporation enhancer were included in each reaction (Additional file 1: Fig. S1b, c) following IDT’s Alt-HDR CRISPR/Cas9 gene editing protocol (https://sfvideo.blob.core.windows.net/sitefinity/docs/default-source/protocol/homology-directed-repair-using-the-alt-r-crispr-cas9-system-and-hdr-donor-oligos.pdf?sfvrsn=47121607_14). The selection of monoclonal mutant populations was performed by doing a single-cell seeding via limiting dilution in 96-well cell culture plates. Validated clones were screened via Sanger sequencing of the *ROS1-SCL34A2* cDNA synthesized with SuperScript™ III First Strand Synthesis kit (ThermoFisher Scientific) and the successfully edited ones were expanded (Additional file 1: Fig. S1d, e). Before proceeding with the functional characterization of the cell lines; the top 5 loci predicted by CHOCHOP for each gRNA sharing the highest homology with the targeted *ROS1* loci were Sanger sequenced to screen for potential CRISPR off-target effects and confirm the presence of the single-nucleotide variants (SNVs) of interest.

### Drug screening

Crizotinib, ceritinib, lorlatinib, ceritinib and repotrectinib were selected based on current approval status by the FDA and EMA, or previous use in clinical trials including *ROS1* + NSCLC. Drug screening on cell lines in 2D monolayers and 3D spheroids was performed at the DrugVision.AI automated screening facility of the University of Antwerp, Belgium. A pre-validated drug screening pipeline was used, for which a detailed protocol is available in the Journal of Visualized Experiments [29]. For 3D spheroids, cells were grown in extracellular matrix domes (Cultrex type 2, Bio-Techne Ltd) for at least two passages to adapt them to 3D culturing conditions as described by Compte et al. [29].

Next, 4-day-old spheroids were harvested from ECM drops using the Cultrex Organoid Harvesting Solution (Bio-Techne Ltd), collected in a 15 mL tube coated with 0.1% BSA/PBS, washed, and resuspended in medium. Next, the number of spheroids was quantified using imaging and diluted in full medium supplemented with 4% Cultrex at a concentration of 4000 organoids / mL. Next, 50µL of this solution was dispensed into a 384-well ultra-low attachment microplate (Corning, #4588) using the OT-2 pipetting robot (Opentrons) in a cooled environment. Thereafter, the plate was centrifuged (100 rcf, 30 s, 4 °C) and incubated overnight at 37 °C. For 2D drug screening, 750 cells were seeded in 384-well optical microplates (Corning, #3764) 24 h before the administration of TKIs.

All drugs and fluorescent reagents were added to the plate using the Tecan D300e Digital Dispenser and dissolved in either DMSO or 0.3% Tween-20/H2O. Cytotox Green (60 nM / well, Sartorius, DMSO) was uses as fluorescent cell death marker and Staurosporine (2 µM, Tocris Bioscience, DMSO) as positive control. For each drug, a 7-point logarithmic titration was dispensed (1 – 5000 nM) and DMSO concentrations were normalized to the same level in each well (< 1%). Brightfield and green fluorescence whole-well images (4 × objective) were taken every 24 h with the Tecan Spark Cyto set at 37 °C / 5% CO2 for 5 days.

### Image and data analysis

Images of 2D monolayers were analyzed using the label-free 2D detection module of the Spark ImageAnalyzer v1.2 (Tecan). Images and data were analyzed with the Orbits® label-free organoid detection module[30]. Viability (V) was quantified as Total Brightfield Organoid Area – Total Green Area and used to calculate the Normalized Growth Rate (NGR):

$$G=\frac{V\left(x\right)- V(0)}{V(0)}$$

$$if\, G>0: NGR={G}_{drug}/{G}_{medNeg}$$

$$if\, G<0: NGR={G}_{drug}/{G}_{medPos}$$

$$NGR={\text{clip}}({\text{NGR}}, [-1, 1])$$

where V(0) is the viability at timepoint 0, V(x) is the viability at timepoint x, Gdrug is the G corresponding to the drug treated condition, GmedPos is the median G of the positive control and GmedNeg is the median G of the vehicle control. Based on the NGR values, the drug effects can be classified as: > 1, proliferative effect; = 1, normal growth as in negative control; = 0, complete growth inhibition; = -1, complete killing as in positive control.

The dose–response relationship was modeled using the Growth Rate (GR) equation.The initial parameter guesses were optimized and derived from Hafner et al. and the GRcalculator tool. The detailed methodology can be found on: https://bioconductor.org/packages/release/bioc/manuals/GRmetrics/man/GRmetrics.pdf. The resulting GR equation is

$$GR=GRinf+(1-GRinf)(\frac{1}{{1+\left(\frac{c}{GEC50}\right)}^{{h}_{GR}}})$$

where GRinf is the response at infinite concentration, GEC50 is the concentration that produces half the maximum possible effect, h_GR is the Hill coefficient, determining the steepness of the curve, and c is the concentration. Next, the Python SciPy library’s ‘curve-fit’ function was employed to fit the GR model to the observed data for each biological replicate. Initial guesses for GRinf, GEC50, and h_GR were set to 0.1, median concentration and 2, respectively. Residual errors between observed and predicted responses were calculated for each data point using the Root Mean Square Error approach. Points exhibiting an error greater than 2.5 times the mean error and an absolute error greater than 0.25 were deemed outliers and the model was refitted to this refined dataset. The following metrics were derived from the fitted curve: NGR50 as the concentration at which the response is 0.5 and NGR_AOC_1_fitted_n as the area over the curve up to y = 1, normalized to the maximum area.

### Immunoblotting

5.10^5^ cells were seeded in 6-well plates and treated 24 h later during 72 h with TKIs dissolved in DMSO. Total protein was collected using RIPA lysis buffer (Thermo Fisher Scientific) supplemented with a tablet of PhosSTOP (Roche) and cOmplete (Roche) per 10 ml of buffer. Protein quantification was performed using the Pierce BCA Assay (ThermoFisher #23,227) and the VICTOR Nivo plate reader (Perkin Elmer) following the manufacturer’s instructions. 10 µg of total, denatured protein were loaded in a 4–12% PAGE gel (Invitrogen) initially during 10 min at 100 V followed by 50 min at 200 V. Proteins were then transferred to a PDVF membrane, which was afterwards blocked with milk powder dissolved in TBST buffer 1,5% (w/v). Eventually, membranes were incubated with 1:2000-diluted primary antibodies anti p-ROS1 Tyr2274 (Cell Signaling #3078), anti ROS1 (Clone D4D6 Cell Signaling #3287), anti p-Erk 1/2 Thr202/Tyr204 (Cell Signaling #4370), anti Erk 1/2 (Cell Signaling #9102) and anti GAPDH (Cell Signaling #5174). As a secondary antibody, HRP-conjugated goat-anti-rabbit was used (Cell Signaling #7074). Finally, membranes were developed using SuperSignal™ West Femto Maximum Sensitivity Substrate (Thermo Scientific #34094). The resulting images were quantified using ImageQuant TL 1D version 8.2 (General Electric). Final western blot images were merged and brightness/contrast-adjusted using ImageJ v.1.54f software (Fiji).

### Statistical methods

The reported data is the result of combining 4 biological replicates for the drug assays of 2D-cultured cells and the combination of 3 technical replicates in the drug screening of spheroids. 2-way ANOVA using GraphPad Prism v8 was used to study differences in AOC between wild type and mutant cells. Dunnett’s multiple comparisons test was performed to determine differences between wild type and mutant cell lines. The chosen alpha value was α = 0.05. Normality and homocedasticity were assumed to perform the 2-way ANOVA.

## Results

### Generation and validation of ROS1-mutant HCC78 cell lines

In this study we selected three ROS1 kinase domain point mutations that confer TKI resistance through different mechanisms and we introduced them in the translocated *ROS1* allele of HCC78 cells through homology-directed DNA repair (HDR) mediated by CRISPR/Cas9 technology. The first mutation modelled is ROS1 p.G2032R, known as a solvent front mutation. It is the most prevalent among patients presenting relapsed disease and is only treatable to some extend with the last-generation TKI repotrectinib. Secondly, we generated a HCC78 ROS1 p.L2026M mutant line, affecting the gatekeeper domain of the kinase. Finally, the ROS1 p.S1986Y mutation was introduced; known to over-activate kinase activity. The cleavage-inducing potential of each designed guide RNA (gRNA) was assessed by fragment analysis. Efficiency is reflected by the number and abundance of alleles that acquired genomic insertions or deletions (indels) as a result of the non-homologous end-joining DNA repair pathway upon cleavage (Additional file 1: Fig. S1b, c). After monoclonal mutant selection, the ROS1 L2026M clone showed a homozygous mutation pattern, while p.G2032R and p.S1986Y showed heterozygous genotypes. Thus, knock-in of the G2032R and S1986Y mutants in the translocated *ROS1* allele was validated through sequencing cDNA using nested PCR (Additional file 1: Fig. S1d, e). Eventually, successful editing of one of the two translocated *ROS1* alleles was demonstrated in both HCC78 ROS1 G2032R and S1986Y clones.

### Repotrectinib shows the highest inhibitory activity across *ROS1* mutations in 2D culture

**Fig. 1:**
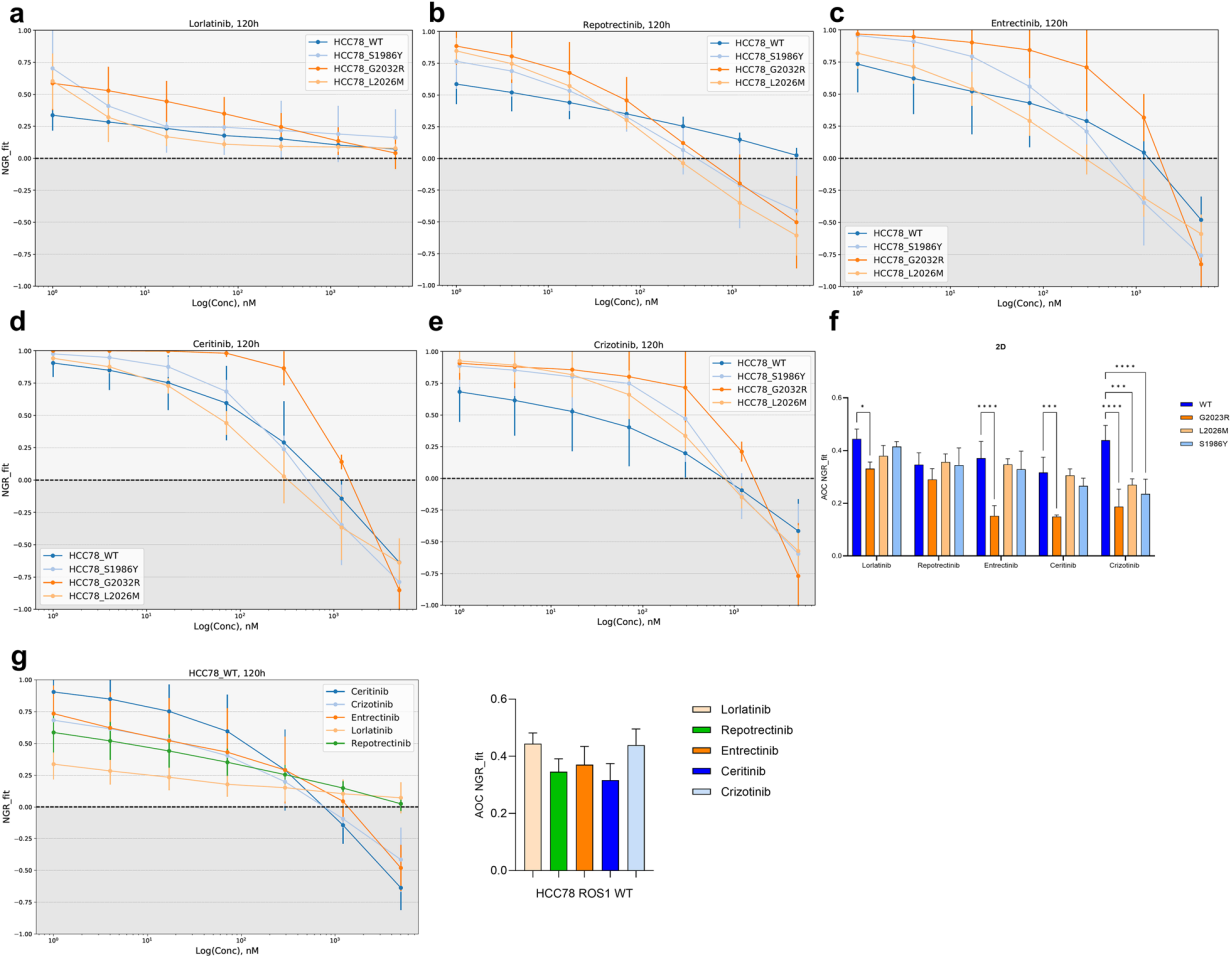
Cellular response in 2D cell culture:** a** Normalized growth rate (NGR_fit) curves obtained by treating the 4 cell lines in 2D with lorlatinib. **b** Repotrectinib. **c** Entrectinib **d** Ceritinib and **e** Crizotinib. **f** Summary of the area over the curve (AOC) values for all treatments and cell lines. ROS1^G2032R^ mutant cells were significantly resistant to lorlatinib (p = 0.01), entrectinib (p < 0.001), ceritinib (p < 0.001) and crizotinib (p < 0.001). ROS1^L2026M^ showed a significant resistance towards crizotinib (p) as well as ROS1^S1986Y^ cells when treated in 2D culture (p). **g** NGR_fit curves and bar plot summarizing AOC values in 2D-treated ROS1 wild type cells. No significant differences in AOC across different TKI treatments were detected. Results are the summary of 4 biological replicates combined

Firstly, we wanted to explore the response towards the FDA- and EMA- approved TKIs to treat *ROS1* + NSCLC like crizotinib, entrectinib and lorlatinib. Ceritinib and repotrectinib were also included in the study, whose activity has been also positively reported against *ALK*-rearranged tumors, a gene that shares a high degree of homology with *ROS1* [31]. The monoclonal HCC78 ROS1 mutant lines G2032R, L2026M, S1986Y and the parental HCC78 ROS1 wild type line were cultured as a monolayer (2D) and treated for up to 120 h with the formerly mentioned TKIs at concentrations ranging from 1 nM to 5 µM. The resulting dose–response curves reflecting the normalized growth rate (NGR) are depicted in Fig. 1. The NGR is a variable used to assess drug-induced changes in cell growth rate. Its values range from − 1 to + 1, where a cytostatic response is reflected by values between + 1 and 0, cytotoxicity between 0 and − 1 and complete lethality when NGR = -1 [27]. The area over the curve (AOC) values with their standard deviation depicted by the 2D and 3D NGR_fit curves are summarized in Table 1. Overall, AOC values observed in 2D cell culture treatments are higher than in 3D cell culture, highlighting an increased refraction towards TKIs when cells are cultured in a monolayer. The complementary half-maximum normalized growth rate (NGR50) values (nM) can be found in Supplementary table 1.

**Table 1:** Area over the NGR_fit curves (AOC)

		Area over the curve (AOC) defined by each NGR_fit plot				
		Lorlatinib	Repotrectinib	Entrectinib	Ceritinib	Crizotinib
2D	Wild type	0.44 ± 0.04	0.34 ± 0.04	0.37 ± 0.06	0.31 ± 0.06	0.43 ± 0.06
	G2032R	0.44 ± 0.02	0.29 ± 0.04	0.15 ± 0.04	0.15 ± 0.01	0.15 ± 0.01
	L2026M	0.38 ± 0.04	0.37 ± 0.03	0.35 ± 0.02	0.31 ± 0.02	0.31 ± 0.02
	S1986Y	0.42 ± 0.02	0.34 ± 0.06	0.33 ± 0.07	0.26 ± 0.03	0.26 ± 0.03
3D	Wild type	0.63 ± 0.04	0.56 ± 0.04	0.52 ± 0.01	0.51 ± 0.01	0.45 ± 0.01
	G2032R	0.11 ± 0.1	0.08 ± 0.09	0.26 ± 0.06	0.18 ± 0.003	0.24 ± 0.06
	L2026M	0.45 ± 0.04	0.25 ± 0.03	0.26 ± 0.02	0.43 ± 0.003	0.54 ± 0.03
	S1986Y	0.56 ± 0.03	0.55 ± 0.04	0.51 ± 0.01	0.51 ± 0.02	0.44 ± 0.02

Summarized AOC values obtained from all the treatments in 2D and 3D cell culture

We observed two different patterns of response among the different TKIs that were tested. On one hand, lorlatinib elicited an early cytostatic response in all 4 cell lines at lower doses, as depicted by the flattened curves that acquired NGR values around 0 (Fig. 1a). On the other hand, the remaining TKIs triggered a cytotoxic cellular response at higher doses (Fig. 1b–e). Concerning the impact of the mutations studied, the ROS1^G2032R^ cell line exhibited the strongest resistant phenotype across all treatments in 2D culture, maintaining the lowest AOC profiles of all 4 tested cell lines. The analysis of the area over the curve (AOC) values depicted by this cell line was significantly higher in all treatments except repotrectinib when compared to the AOC corresponding to the wild type cell line. Thus, a clear resistance towards lorlatinib (p = 0.01), entrectinib (p < 0.001), ceritinib (p < 0.001) and crizotinib (p < 0.001) was detected. The ROS1^L2026M^ variant conferred a significant resistant phenotype against crizotinib (p < 0.001) according to the AOC metrics. ROS1^S1986Y^ HCC78 cell line reflected comparable sensitivities to TKIs to wild type cells except when treated with crizotinib, whose AOC was significantly lower than the area depicted by wild type cells (p < 0.001). In consequence, repotrectinib emerged as the single TKI able to overcome the effect of the three resistance-conferring variants in 2D cell culture.

### Levels of phosphorylated ROS1 upon TKI treatment confirm the results of the 2D cell viability assays

**Fig. 2:**
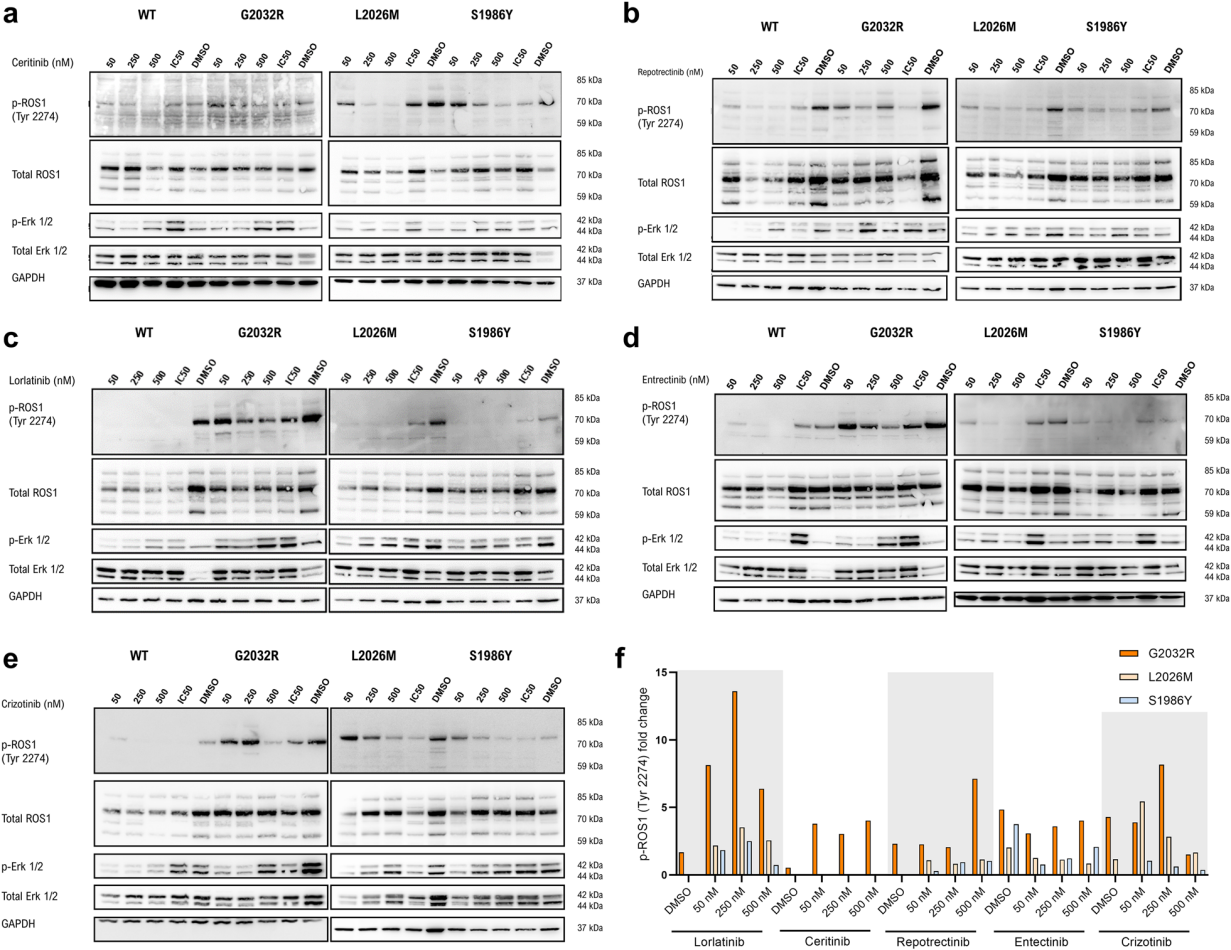
Immunoblotting of treated cell lines. Western blot of depicting p-ROS1 (Tyr 2274), the different ROS1 isoforms expressed by HCC78 cells (total ROS1), p-Erk 1/2 (Thr 202 / Tyr 204), total Erk 1/2 and GAPDH of ceritinib (**a**) IC50s: WT = 1.5 nM, G2032R = 179.3, L2026M = 44.18, S1986Y = 101.6; repotrectinib (**b**) IC50s: WT = 14 nM, G2032R = 318 nM, L2026M = 165.3 nM, S1986Y = 56.4 nM, lorlatinib (**c**) IC50s: WT = 1.13 nM, G2032R = 59.52 nM, L2026M = 2 nM, S1986Y = 1.81 nM; entrectinib (**d**) IC50s: WT = 13.52 nM, G2032R = 207 nM, L2026M = 18.5 nM, S1986Y = 15.5 nM and crizotinib (**e**) IC50s: WT = 112 nM, G2032R = 1692 nM, L2026M = 601 nM and S1986Y = 214.4 nM. **f** Bar plot reflecting the fold change of normalized p-ROS1 (Tyr 2274) levels relative to ROS1 wild type across TKI treatments in 2D culture

In order to verify that the cytostatic and/or cytotoxic effect induced by the TKIs is caused by the targeted inhibition of the phosphorylation of ROS1 (tyrosine 2274), we treated the three mutant clones and the wild type line all grown in 2D with 50 nM, 250 nM, 500 nM and IC50 (mutation-tailored) with the 5 TKIs aforementioned. We next blotted the levels of phosphorylated ROS1, Erk 1 and 2 to gain insights about the state of MAPK pathway upon ROS1 inhibition in HCC78 cells (Fig. 2). Interestingly, the western blot assay revealed that the four clones express 5 different isoforms of the ROS1 protein instead of the three reported by the antibody supplier (85, 70 and 59 kDa). These two newly identified isoforms have a molecular weight around 65 and 75 kDa approximately. However, the isoform that contains the tyrosine 2274, phosphorylated upon kinase activation (thereafter referred as p-ROS1), is present only in the 70 kDa isoform.

As shown in Fig. 2, crizotinib suppressed the phosphorylation of ROS1 wild type cells although it failed to do so in the mutant cell lines since the p-ROS1 band was more intense in ROS1^G2032R^-treated cells, followed by ROS1^L2026M^ and finally ROS1^S1986Y^, in which a partial inhibition of the phosphorylation could be observed. This pattern was consistent across the different TKI treatments although with differences in magnitude.

Repotrectinib clearly inhibited the phosphorylation of wild type ROS1 kinase and to a lesser extent, the mutants ROS1^L2026M^ and ROS1^S1986Y^; in which a band can be observed even when cells are treated at 500 nM (Fig. 2b). ROS1^G2032R^ cells displayed the highest p-ROS1 levels and only when treated with their corresponding IC50 of 1.24 µM, the phosphorylation could be partially blocked.

Lorlatinib and entrectinib inhibited more strongly the autophosphorylation of ROS1^L2026M^ and ROS1^S1986Y^ but p-ROS1^G2032R^ levels did not substantially decrease. Finally, ceritinib treatment resulted in the partial inhibition of ROS1^WT^, ROS1^L2026M^ and ROS1^S1986Y^ phosphorylation; in a less efficient manner than the former TKIs in this study. In concordance with the other treatments, p-ROS1^G2032R^ levels were the highest across conditions, confirming the strong impact of the variant in TKI resistance (Fig. 2c–e). All these observations are quantitatively shown in Fig. 2f, reflecting the fold change of normalized p-ROS1 levels relative to wild type cells.

With regard to p-Erk 1/2 levels, it is important to notice a recurrent pattern across treatments. We observed that at 50 nM and 250 nM, p-Erk1/2 levels are low; but when the concentrations are increased to 500 nM or higher in case of some IC50s, p-Erk 1/2 levels also raise. This increase in a dose-dependent manner might point towards an activation of the MAPK pathway through a cytotoxic-mediated apoptosis. Although the quantified levels of p-ROS1 of the imaged membranes did not significantly change (Additional file 1: Fig. S2), a trend can be clearly observed. Nevertheless, the western blots offer a qualitative overview of the activity of TKIs that complements the results obtained in the performed drug screenings.

### TKIs exert higher activity in ROS1^mutant^ spheroids

**Fig. 3:**
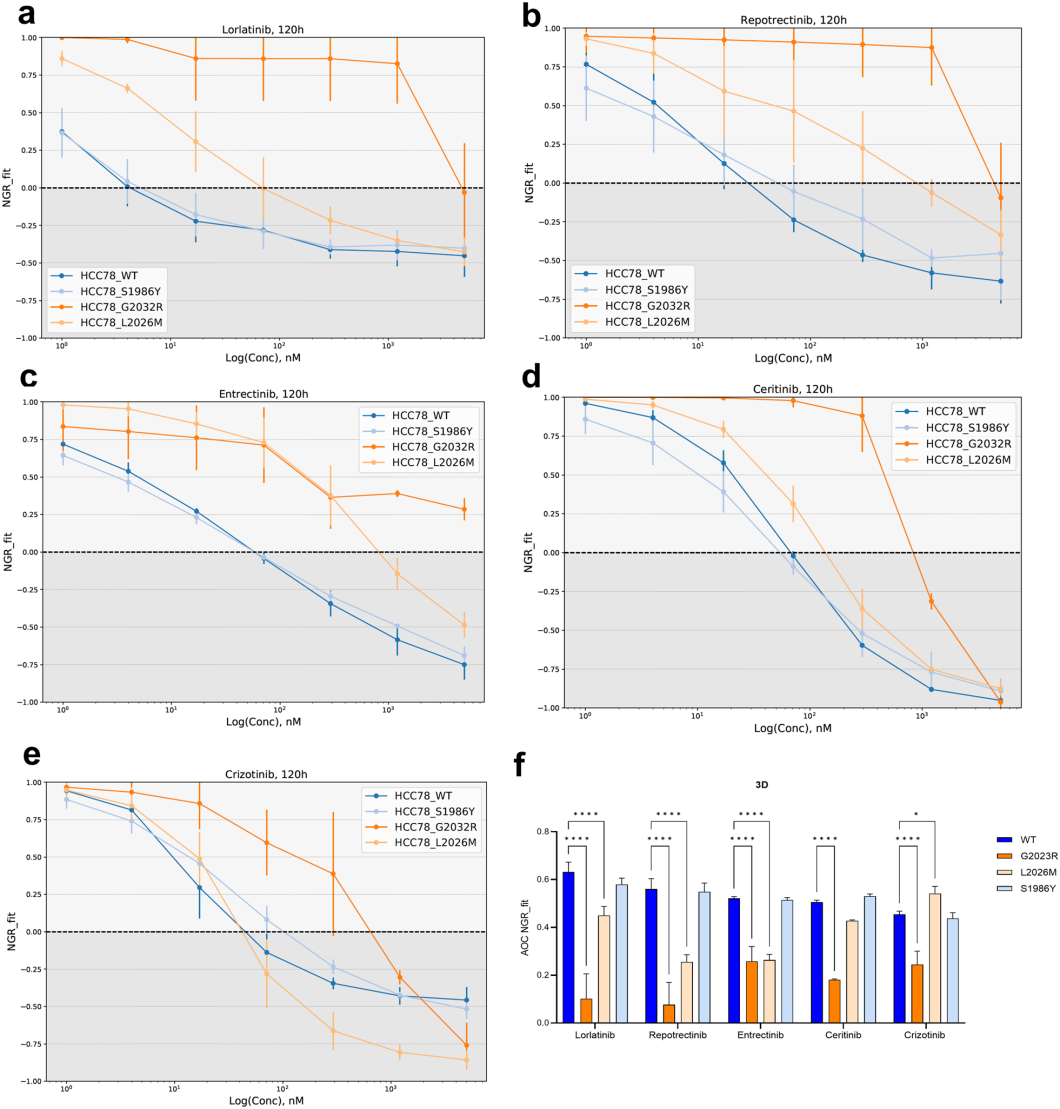
Cellular response in 3D cell culture. **a** NGR_fit curves obtained by treating the 4 cell lines in 3D with lorlatinib. **b** Repotrectinib. **c** Entrectinib **d** Ceritinib and **e** Crizotinib. **f** Summary of the AOC values for all treatments and cell lines revealing a resistant phenotype of ROS1^G2032R^ spheroids towards lorlatinib (p < 0.001), repotrectinib (p < 0.001), entrectinib (p < 0.001), ceritinib (p < 0.001) and crizotinib (p < 0.001). ROS1^L2026M^ spheroids showed resistance against lorlatinib (p < 0.001), repotrectinib (p < 0.001) and entrectinib (p < 0.001) but displayed an increased sensitivity towards crizotinib (p = 0.04). Results are the summary of 3 technical replicates combined

We then generated spheroids from the 4 cell lines and treated them using the same approach as previously described. Interestingly, we noticed that the effect of the introduced variants became more pronounced when culturing cells in 3D, as shown by the separation between the different NGR curves depicted by each cell line. ROS1^G2032R^ mutant spheroids were significantly more resistant towards all the TKIs compared to wild type spheroids: lorlatinib (p < 0.001), repotrectinib (p < 0.001), entrectinib (p < 0.001), ceritinib (p < 0.001) and crizotinib (p < 0.001) according to the AOC values (Figs. 3a–e). ROS1^L2026M^ spheroids were significantly resistant against lorlatinib (p < 0.001), repotrectinib (p < 0.001) and entrectinib (p < 0.001). However, an increased sensitivity towards crizotinib (p = 0.04) was identified. Interestingly, no differences in AOC value were detected upon ceritinib treatment when comparing them to wild type spheroids. ROS1^S1986Y^ spheroids reflected similar TKI sensitivities to their wild type counterparts, indicating the lack of advantage conferred by the S1986Y variant when growing cells in 3D culture. All the AOC values corresponding to each treatment are summarized in Fig. 3f.

**Fig. 4:**
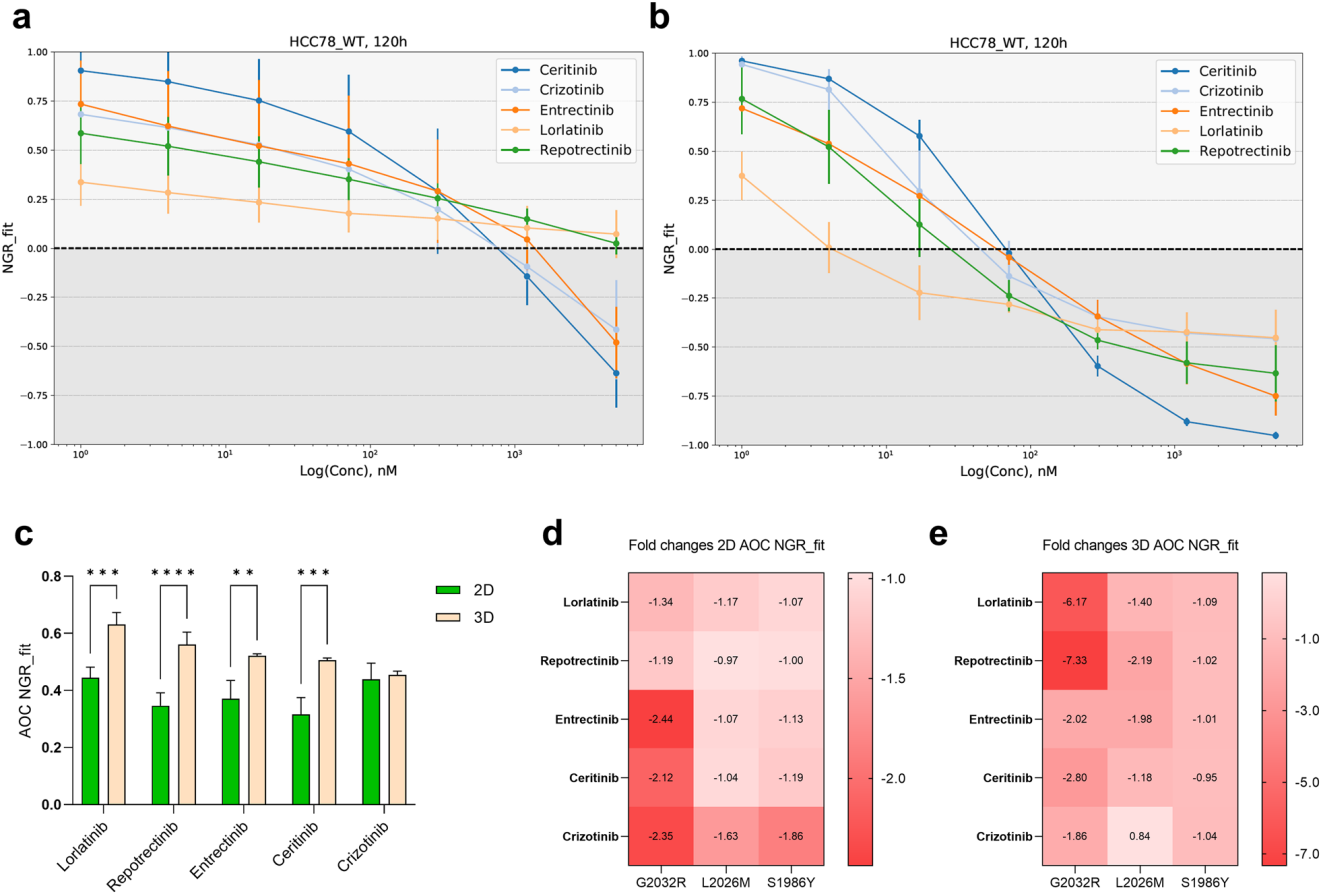
**a** Dose-reponse profile of the 5 TKIs tested in HCC78 wild type cells cultured in 2D and **b** forming spheroids. **c** Summary of AOC values revealing the increased sensitivity of HCC78 spheroids when treated with lorlatinib (p < 0.001), repotrectinib (p < 0.001), entrectinib (p = 0.002) and ceritinib (p < 0.001). **d** Fold changes of the AOC obtained from the NGR_fit curves of mutants versus wild type cell lines and **e** spheroids. The negative signs indicate a decrease in the fold change, whilst positive values reflect an increase in the metric

The cell culture approach determined the response to TKI in HCC78 wild type cells. As shown in the NGR_fit curves in Fig. 4a, b and the plotted AOC values in Fig. 4c, lorlatinib (p < 0.001), repotrectinib (p < 0.001), entrectinib (p = 0.001) and ceritinib (p < 0.001) treatments in 2D cells yielded significantly lower AOC values. This reflects an increased sensitivity of HCC78 cells to TKIs when cultured as spheroids. Additionally, the AOC fold changes were obtained for the three mutant cell lines relative to wild type cells in 2D (Fig. 4d) and 3D cell culture (Fig. 4e). Importantly, the highest AOC fold change in 2D cell culture was observed upon the ROS1^G2032R^ treatment with entrectinib (fold change = -2.44) and crizotinib (fold change = -2.35), reflecting the poor inhibitory activity of ROS1 wild type cells by these TKIs. A similar pattern was observed in 3D culture, in which ROS1^G2032R^ spheroids showed a pronounced refractory phenotype towards all TKIs. Notably, the highest AOC fold changes were observed for newer-generation TKIs repotrectinib (fold change = -7.33) and lorlatinib (fold change = -6.17). However, close inspection indicated that this fold change was not caused by a reduced performance of the TKIs against mutants, but a more potent inhibition of the wild-type lines, which can be seen from the concentration at which the NGR_fit values reach zero (Figs. 1 and 3). Consequently, the increased fold change actually indicates a partially masked TKI response due to the EGFR-dependency in 2D cell culture, which is absent in 3D cell culture.

**Fig. 5:**
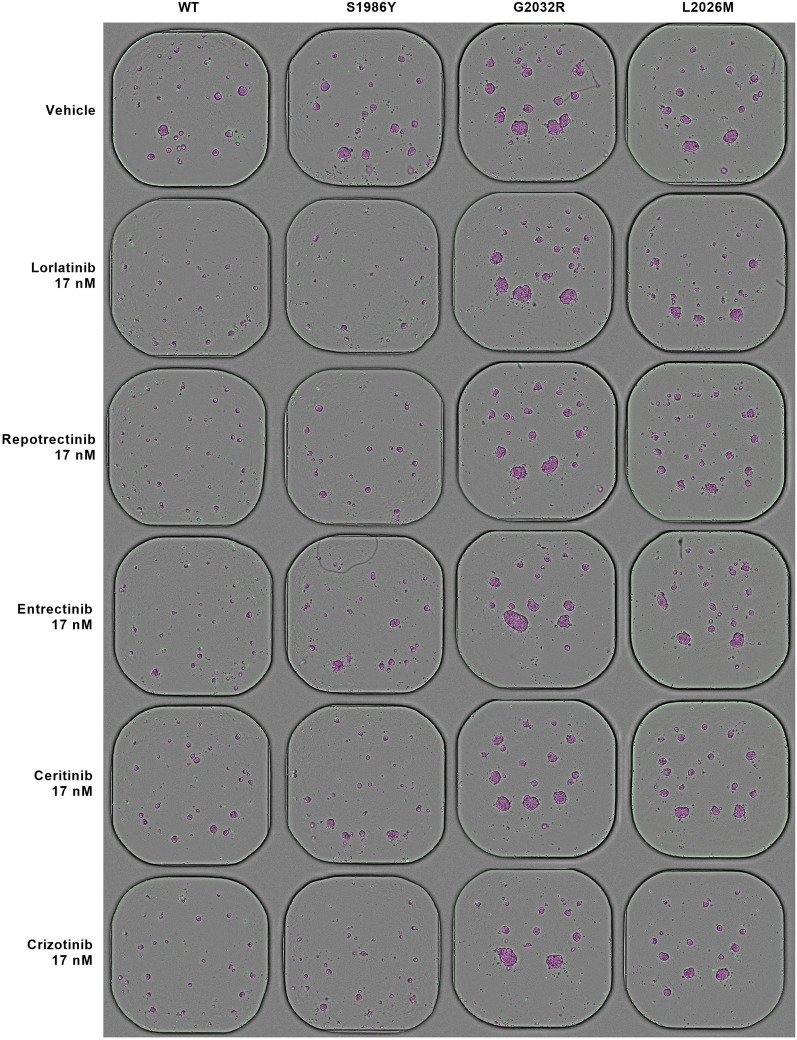
Multiplex TKI screening in spheroids. 120 h post-TKI treatment 17 nM of ROS1 wild type and mutant HCC78 spheroids. Magenta: Orbits^®^ label-free organoid segmentation defining the total spheroid area. Green: fluorescence cytotox green signal staining dead cells

Figure 5 summarizes the morphological features of the spheroids at 120 h of TKI treatment at a concentration of 17 nM together with DMSO only-treated spheroids. This reagent was used as a vehicle to deliver the compounds to cells. The 17 nM concentration was chosen as a balance point between effective against resistant mutants and tolerble by wild type spheroid cultures. Phenotypically, wild type and mutant spheroids were similar in absence of TKIs. However, when incorporating the drugs, the differences in spheroid size across genotypes become evident and are concordant with the magnitude of their corresponding AOC values. The developed software allows the individual segmentation of spheroids, each of them labelled with a magenta mask. Additionally, the cell viability dye Cytotox Green binds the DNA of cells whose plasma membrane integrity is lost, thus indicating cell death.

## Discussion

Developing refined pre-clinical disease models is currently one of the major challenges, especially when it comes to approaching conditions characterized by a low prevalence. This is the case for *ROS1* + NSCLC, a malignancy that is also associated with a remarkable variability in terms of patient response towards TKI treatment. Thus, in this study we use for the first time CRISPR/Cas9 technology to introduce single nucleotide variants of interest in a *ROS1* + patient-derived cell line, preserving the genetic background of the cell line and the expression levels of the translocated *ROS1* alleles. Such features increase the representative value of the model for the *SLC34A2-ROS1*-rearranged NSCLC molecular subclass of tumors. In addition, the use of an automated script that analyzes bright field images to monitor the response to TKIs allows a reproducible and scalable system that is complemented with a morphological assessment of 2D or 3D cell cultures thanks to the live cell imaging system [30]. Furthermore, the implementation of the AOC as a variable to assess the activity of TKIs holds several advantages in comparison with traditional cell viability assays. Firstly, it allows the profiling of cytostatic activity of drugs in addition to a cytotoxicity assessment. This feature can be time-monitored by just imaging the plates at the desired time points, providig a clear overview of the cellular response. Secondly, the use of the same metric for 2D and 3D cell culture facilitates the direct comparison between culture systems.

With the incorporation of the CRISPR/Cas9 technology to study variants identified in the clinical setting, a new refinement strategy towards pre-clinical models is established. The majority of pre-clinical studies that focus on assessing the activity of TKIs on cells expressing ROS1 fusions carrying resistance mutations are based on transformed Ba/F3 cells. Although they are a good tool to quickly profile the TKI activity, Ba/F3 cells lack the lineage and mutational landscape of *ROS1* + NSCLC cells. Therefore, new approaches are needed to generate more representative disease models. In this project we describe the optimized protocol to obtain monoclonal mutant cell lines by delivering the CRISPR/Cas9 in ribonucleoprotein (RNP) form through nucleofection. The experimental pipeline required a thorough optimization concerning the electroporation parameters and single-cell based mutant selection as depicted in Additional file 1: Fig. S1; with the advantage that once the right conditions are determined, the protocol can be directly implemented in cell lines of a same lineage. Exploring the role of rare variants is the next challenge, particularly in *ROS1* + NSCLC, in which the access to tumor samples remains difficult due to the low prevalence of *ROS1* rearrangements among lung adenocarcinoma patients. With this project we set the experimental proof-of-concept to approach resistance-conferring variants in the first *ROS1* + reported cell line: HCC78. Nevertheless, new patient-derived cell lines have been established within the last years such as the CUTO lines [32]. They comprise a collection of different ROS1 fusions that allow the evaluation of the role of the *ROS1* fusion partner and inter-patient variability in the biology of neoplastic cells. Thus, translating the experimental approach described in this study into the aforementioned new models will provide an overview of the TKI sensitivity profiles defined by the mutant cell lines and eventually guide clinical decision-making. Furthermore, the advantage of culturing HCC78 forming spheroids allowed the subtraction of the increased EGFR signaling background observed in 2D culture which renders HCC78 cells less sensitive to TKIs in 2D cell culture. Thus, only the effect of the resistance-conferring variants explained the clear differences in TKI sensitivity across the mutant spheroids. A striking observation concerns the significant increase of the AOC versus wild type cells depicted by the ROS1^L2026M^ spheroids treated with crizotinib. A priori, it might seem a contradictory result based on the clinical evidence that confirms the role of the L2026M variant as resistance-conferring. Nonetheless, one should consider two aspects: firstly, the presence of the L2026M variant in both *ROS1*-rearranged alleles compared to the G2032R and S1986Y lines, in which the point mutation was introduced only in one allelle. Secondly, the fact that high ROS1 signaling levels exceeding a certain threshold trigger apoptotic cell death, as demonstrated by Ogura et al.[33] Therefore, the ROS1^L2026M^ line presumably synthetizes a higher amount of the resistant ROS1 fusion which, not being inhibited by crizotinib, could result in higher ROS1 activation levels. Coupling these scenarios could explain why it was only detected in 3D culture; a system where HCC78 cells become more dependent on the ROS1-mediated signaling.

The drug sensitivity patterns obtained in vitro are aligned with the clinical observations from two points of view. Firstly, the superiority of last generation TKIs like repotrectinib and lorlatinib compared to first generation compounds such as crizotinib and entrectinib [10, 34–36]. Recent compounds have been structurally improved to increase kinase specificity and to bypass resistance mutations [37]. Secondly, we also show that the G2032R ROS1 variant results in the strongest drug-refractory phenotype across compounds regardless of the culture system used. Although we determined lower AOC values than wild type for ROS1^L2026M^ HCC78 cells and in some cases, ROS1^S1986Y^ cells, only some of them were significant. However, the trends regarding the dose–response curves are consistent with previous in vitro assays using transformed Ba/F3 cell lines [38]. Interestingly, the mutation ROS1^S1986Y^ did not seem to confer a strong advantage to HCC78 cells upon TKI treatment except when treated with crizotinib in 2D. These observations are in contrast with results in Ba/F3 cells expressing a EZR-ROS1^S1986Y^ fusion, where S1986Y mutants confered resistance to ceritinib as well [39]. A potential explanation here could be the *ROS1* fusion partner gene, which combined with a more representative cellular background, might result in different protein expression levels and subcellular localization, ultimately modulating the oncoprotein dependency of neoplastic cells.

When assessing the shift from 2 to 3D culture system towards the TKI response, we observe a significant increase in AOC values for wild type models, suggesting a reduction of the EGFR-dependency already described in HCC78 cells. Importantly, we noticed different patterns between 2 and 3D cultures concerning the AOC fold changes in the mutant versus wild type lines. In 2D culture, the biggest fold changes in ROS1^G2032R^ mutant cells were observed for crizotinib, ceritinib and entrectinib treatments. In contrast; the same line cultured in 3D revealed that lorlatinib and repotrectinib elicited a higher resistant response compared to ROS1 wild type spheroids. These apparently contradicting patterns might be explained by how HCC78 wild type cells respond to TKIs in both culture systems. Crizotinib was the only compound that did not significantly change the AOC values in wild type cells cultured in 2D versus 3D systems. All other TKIs showed significantly increased AOC values in spheroids. Consequently, next-generation TKIs such as repotrectinib and lorlatinib depict stronger fold changes in 3D for mutant lines due to a stronger inhibition ofthe wild type spheroids, rather than a worse inhibition of the mutants, As the baseline inhibition is stronger compared to first-generation TKIs, the difference to a refractory line becomes more pronounced.. Collectively, our results indicate that the impact of resistance-conferring variants in 2D-cultured HCC78 cells can be partly masked by the constitutively activated EGFR pathway.

The major clinical implications that can be retrieved from our study are mainly directed towards *SLC34A2-ROS1* rearranged lung adenocarcinomas and, according to our spheroid drug assay, all the 5 TKIs evaluated effectively targeted ROS1 wild type and S1986Y mutant cells. However, patients presenting the solvent front G2032R or the gatekeeper L2026M mutations might benefit from treated with lorlatinib or repotrectinib if possible. Nevertheless, remarkably higher doses of such TKIs would be needed to effectively induce the death of the mutant tumor cells. However, this increase in dose might surpass the therapeutically tolerable window, rendering the therapy non-applicable.

Overcoming the effect of resistant mutations remains a clinical challenge. New compounds with promising inhibitory activity such as NVL-520 position themselves as excellent first-line TKIs upon the detection of aggressive variants like G2032R or L2026M. Nevertheless, a more comprehensive characterization of the molecular alterations of *ROS1* + NSCLC is needed to identify new druggable targets. By doing so, novel combinatory approaches could be established that would enrich the treatment options for patients facing TKI refractory tumors.

An important aspect to be noticed involves the impact of the cell culture method used to perform drug viability assays. As previously reported by Gong et al., the dependency of ROS1 fusions in HCC78 cells was enhanced when cultured in presence of the polymer gellan gum [24]. In our study, by generating spheroids through an independent approach using cultrex, we could determine an increase in drug sensitivity; indicating that cell-to-cell interactions are likely to modulate the oncogenic signaling through the EGFR-dependency in 2D culture of HCC78 cells. Thus, this phenomenon should be considered during the experimental design process involving HCC78 cells, particularly when aiming to compare results of 2D drug assays performed in different cell lines. It is also worth noticing the presumable dose-dependent activation of the MAPK pathway upon TKI treatment in HCC78 cells. The mechanism behind this finding in 2D cell culture might be the switch towards an EGFR-mediated MAPK activation upon ROS1 inhibition. Nonetheless, this hypothesis should be further explored in new experiments.

This study has some limitations. Firstly, the use of a single cell line to explore the impact of kinase point mutations; future experiments should be performed in other *SLC34A2-ROS1*-rearranged NSCLC cell lines before extrapolating the conclusions of the drug assays to the clinical setting. Secondly, the lack of representation of other *ROS1* rearrangements. Further studies implementing this combined approach should be performed in patient-derived cell lines that harbor different *ROS1* rearrangements to study the contribution of the *ROS1* fusion partners. Thirdly, the use of NGR-based metrics and Western blot are valuable surrogate markers to monitor drug response. However, complementary experiments elucidating the specific cell death mechanism behind the cytotoxic activity of TKIs would be beneficial. Another relevant limitation are the potential off-target mutations caused by the CRISPR/Cas9 technology. Although we sequenced the 5 loci most susceptible to be targeted by each guide RNA (not shown), we cannot exclude the existence of indels introduced by the gene editing tool. However, the similarity between in vitro assays and patient data regarding the aggressiveness of each the mutations we studied indicates that any potential off-target mutation might have a low impact in terms of drug response. However, performing drug screenings and other functional testing in independently knocked-in lines harboring the same point mutation would highlight the converging mechanisms of drug resistance attributed to the variants. To sum up, our experimental setting holds a remarkable value that is offers a quick and high-throughput drug screening; two important features required to model in vitro newly identified variants with an unknown TKI response profile.

Additionally, further studies should be focused in expanding the collections of ROS1-mutant patient-derived cell lines so that other ROS1 fusion types can be investigated. This will allow a clear definition of the therapeutical implications of different resistance-conferring mutations across the diversity of *ROS1*-rearranged malignancies. Neel and colleagues reported that the ROS1-mediated oncogenic signaling is greatly modulated by the subcellular localization of the ROS1 fusion[2], therefore different *ROS1* translocations harboring the same resistance mutations could result in different sensitivity profiles towards TKIs. Complementary, in vivo studies could also be performed using CRISPR/Cas9-edited cell lines. They indeed offer valuable insights about the efficacy of TKIs in presence of ROS1 point mutations given that they recapitulate the interactions of tumor cells with the stromal compartment. However, we believe that orthotopic murine models could be established based on other cell lines like gene-edited CUTO or ADK-VR2 lines. Since they are novel cell lines and no additional oncogenic mutations have been reported in them, they might be more interpretable candidates.

Taken together, our results point towards the importance of choosing the right sequential treatment path. Upon the detection of *ROS1*-rearranged NSCLC, the first-line treatment would be crizotinib or entrectinib, depending on the presence of brain metastases. However, upon disease progression, a next-generation sequencing (NGS)-based testing of the lesion is encouraged. if kinase point mutations constitute the mechanism of TKI resistance, the variant identified will condition the subsequent treatment lines. In case G2032R is detected, a strong preference towards repotrectinib and lorlatinib should be considered. Nevertheless, known clinical responses for these compounds are low for G2032R, with experimental compounds such as NVL-520 offering more promising results. Additionally, further research to unveil the mechanisms that result in a poor response to immunotherapies beyond the already known low tumor mutational burden (TMB) of *ROS1* + NSCLC [40]. Engaging the immune system in the treatment of such malignancies remains the unresolved subject due to a poor understanding of the tumor biology (Additional file 2).

## Conclusion

In this project we incorporate the use of CRISPR/Cas9 technology to knock-in clinically reported, resistance-causing variants affecting the ROS1 kinase domain in the HCC78 cell line. As a result, three mutant cell lines were obtained, carrying each of them the variants G2032R, L2026M and S1986Y, respectively. Pharmacological assays in 2D and 3D were concordant, highlighting the moderate performance of next-generation TKIs lorlatinib and repotrectinib against the ROS1^L2026M^ and ROS1^S1986Y^, and the low activity against ROS1^G2032R^ mutant cells. Hence, the G2032R conferred the strongest TKI refractory phenotype, followed by L2026M. Notably, the S1986Y elicited resistance towards crizotinib in 2D culture. Moreover, immunoblotting assays confirmed the ROS1-dependence of HCC78 cells observed in the in vitro drug assays through a dose-dependent inhibition of ROS1 phosphorylation. Strikingly, p-Erk 1/2 levels increased in a dose-dependent manner, potentially reflecting a switch towards an EGFR-mediated MAPK activation. In parallel, activated MAPK might also reflect the trigger of apoptosis. These results are primarily applicable to *SLC34A2-ROS1*-rearranged NSCLC, and the fact of respresenting a single cell line constitutes a limitation of the study. Thus, our methodology should be extended to other cell lines harboring different *ROS1* rearrangements to corroborate our findings. In this project, we demonstrate how CRISPR/Cas9 can be implemented to model resistance-conferring mutations and establishes NGR metrics calculated through live cell imaging as an excellent platform to accurately examinate the behavior of spheroids and quantify their response towards treatment. Importantly, given that CRISPR/Cas9 technology refines the modeling of other genetic variants such as nucleotide insertions and deletions (indels) or chromosomal rearrangements, our results can be applied to a wider array of malignancies facing treatment resistance. To sum up, the present combination of techniques implies an innovative approach to accurately define the impact of emerging TKI-resistant variants and ultimately boosting drug development processes in a high-throughput manner.

### Supplementary Information

**Additional file 1: Fig. S1.** Optimization of the CRISPR/Cas9-mediated mutagenesis protocol. **a** Nucleofection efficiencies expressed as percentage of GFP + cells. A combination of different Amaxa 4D Nucleofector programs were tested with three different buffers. **b** Fragment analysis electropherogram of the ROS1 genomic region encoding exon 38. The nucleofection of the RNP containing the gRNA used to introduce the variant resulting in the G2032R mutation had low cleavage efficiency due to inefficient RNP delivery. **c** The incorporation of the electroporation enhancer (IDT) dramatically increased the delivery efficiency of the RNP as shown in the indels introduced in the intended locus. **d** Transcript of the oncogenic fusion expressed in HCC78 cells. To validate the presence of the mutations within the *ROS1* rearranged alleles, a nested PCR protocol was established. A first PCR was done using the *ROS1* fusion cDNA as a template and a forward primer that binds the *SLC34A2* gene to exclusively amplify ROS1-translocated alleles. Since the first PCR product is too long to be Sanger sequenced, a second PCR was done using this long product to amplify only the region harboring the mutation. **e** Sequences of the validated HCC78 *ROS1* mutant clones used in this study. **Fig. S2.** Quantification of the western blot p-ROS1 bands using ImageQuant TL v 8.2 (General Electric). 2-way ANOVA was performed using Dunnett’s multiple comparisons test with GraphPad Prism v8.

**Additional file 2: Table S1.** Metrics of NGR curves. Summarized half-maximum normalized growth rate (NGR50) values obtained from all the treatments in 2D and 3D cell culture (nM).

## Data Availability

All data generated or analyzed during this study are included in this published article (and its supplementary material).
