# Supplementary figures and images for: CRISPR/Cas9-edited ROS1 + non-small cell lung cancer cell lines highlight differential drug sensitivity in 2D vs 3D cultures while reflecting established resistance profiles
Source: J Transl Med. 2024 Mar 3;22:234. doi: 10.1186/s12967-024-04988-0 (PMC10910754; doi:10.1186/s12967-024-04988-0)

## Slide 1
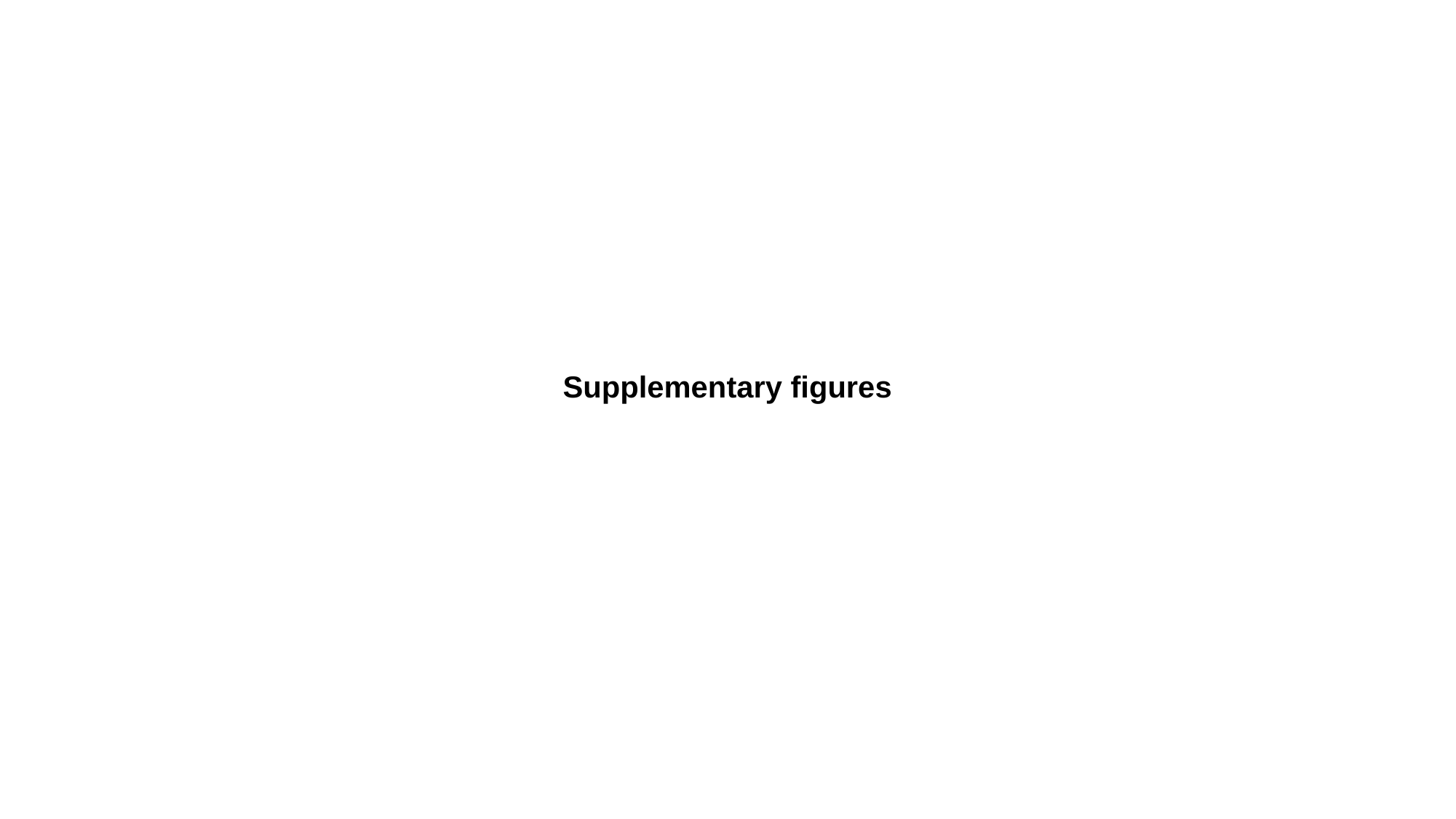

Supplementary figures

## Slide 2
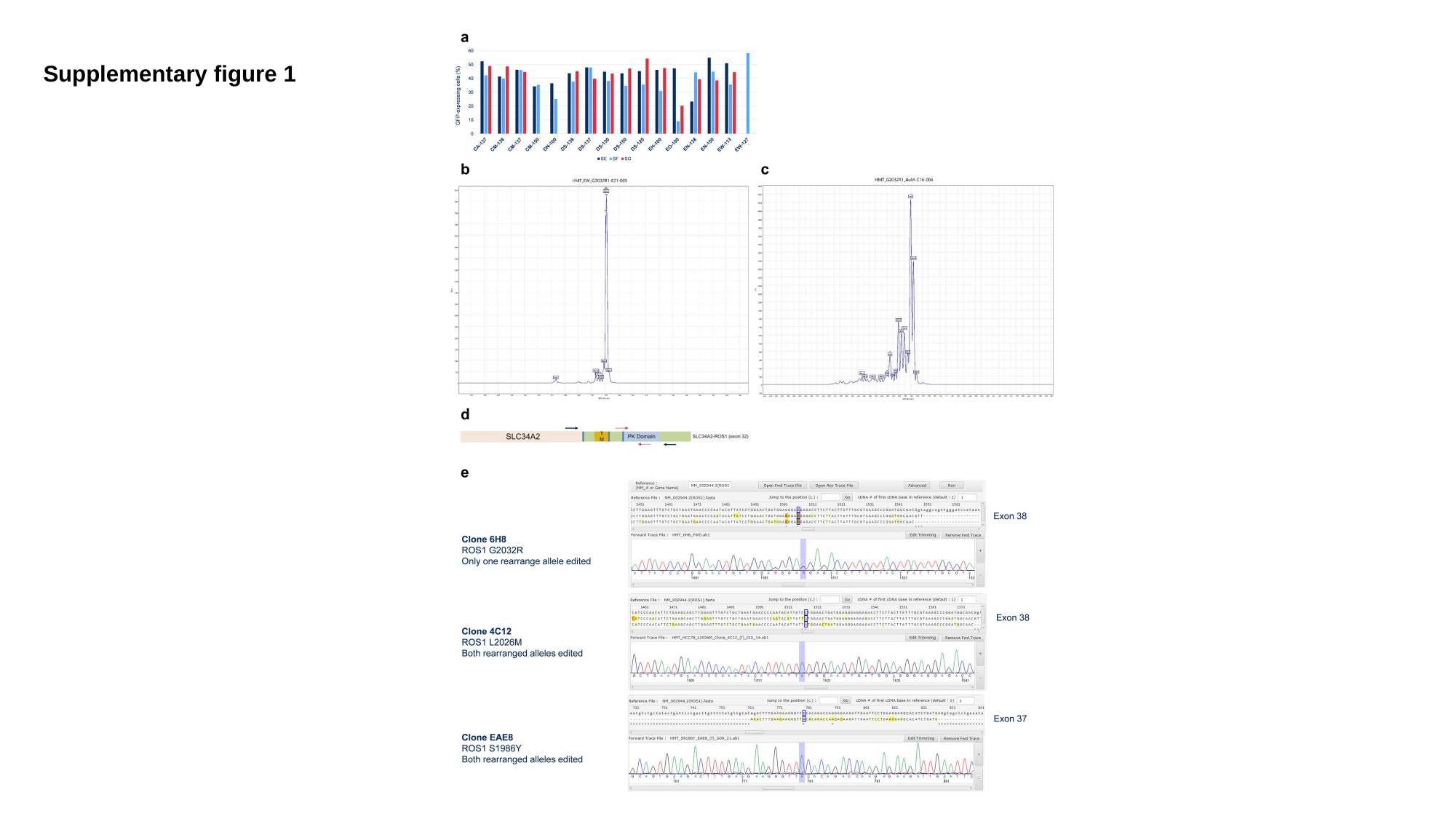

# Supplementary figure 1

## Slide 3
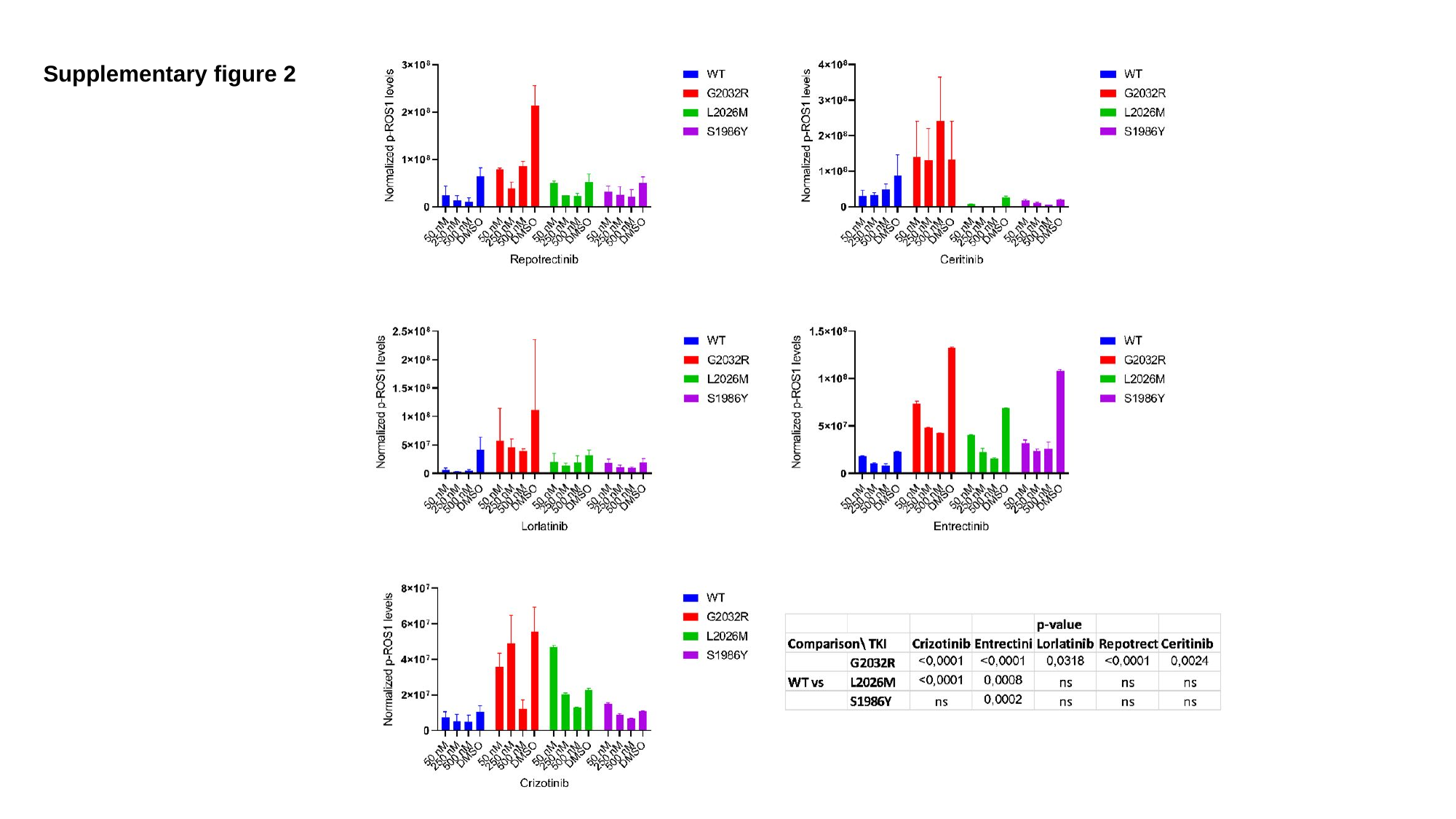

# Supplementary figure 2

Supplement: Supplementary file 1 — Additional file 1: Fig. S1. Optimization of the CRISPR/Cas9-mediated mutagenesis protocol. a Nucleofection efficiencies expressed as percentage of GFP + cells. A combination of different Amaxa 4D Nucleofector programs were tested with three different buffers. b Fragment analysis electropherogram of the ROS1 genomic region encoding exon 38. The nucleofection of the RNP containing the gRNA used to introduce the variant resulting in the G2032R mutation had low cleavage efficiency due to inefficient RNP delivery. c The incorporation of the electroporation enhancer (IDT) dramatically increased the delivery efficiency of the RNP as shown in the indels introduced in the intended locus. d Transcript of the oncogenic fusion expressed in HCC78 cells. To validate the presence of the mutations within the ROS1 rearranged alleles, a nested PCR protocol was established. A first PCR was done using the ROS1 fusion cDNA as a template and a forward primer that binds the SLC34A2 gene to exclusively amplify ROS1-translocated alleles. Since the first PCR product is too long to be Sanger sequenced, a second PCR was done using this long product to amplify only the region harboring the mutation. e Sequences of the validated HCC78 ROS1 mutant clones used in this study. Fig. S2. Quantification of the western blot p-ROS1 bands using ImageQuant TL v 8.2 (General Electric). 2-way ANOVA was performed using Dunnett’s multiple comparisons test with GraphPad Prism v8. [file 12967_2024_4988_MOESM1_ESM.pptx]
