# Supplementary material for: CRISPR/Cas9-edited ROS1 + non-small cell lung cancer cell lines highlight differential drug sensitivity in 2D vs 3D cultures while reflecting established resistance profiles
Source: J Transl Med. 2024 Mar 3;22:234. doi: 10.1186/s12967-024-04988-0 (PMC10910754; doi:10.1186/s12967-024-04988-0)
Supplement: Supplementary file 2 — Additional file 2: Table S1. Metrics of NGR curves. Summarized half-maximum normalized growth rate (NGR50) values obtained from all the treatments in 2D and 3D cell culture (nM). [file 12967_2024_4988_MOESM2_ESM.docx]

|  | | **NGR50 (nM)** | | | | |
| --- | --- | --- | --- | --- | --- | --- |
|  |  | Lorlatinib | Repotrectinib | Entrectinib | Ceritinib | Crizotinib |
| 2D | Wild type | 0.93 +/- 0.97 | 4.07 +/- 1.26 | 784.83 +/- 662.86 | 521.97 +/- 97.82 | 225.47 +/- 217.68 |
|  | G2032R | 346.19 +/- 410.3 | 557.83 +/- 249.83 | 1377.2 +/- 361.1 | 806.53 +/- 229.3 | 990.68 +/- 229.3 |
|  | L2026M | 2.647 +/- 2.54 | 559.25 +/- 550.43 | 384.76 +/- 216.21 | 455.25 +/- 292.26 | 455.25 +/- 292.26 |
|  | S1986Y | 1.44 +/- 2.02 | 248.37 +/- 90.89 | 543.21 +/- 542.69 | 659.84 +/- 629.69 | 732.35 +/- 152.89 |
|  | | | | | |  |
|  |  |  |  |  |  |  |
| Spheroids | Wild type | 3.36 +/- 4.3 | 11.14 +/- 6.79 | 56.04 +/- 3.96 | 65.59 +/- 3.36 | 12.48 +/- 3.25 |
|  | G2032R | 2996.38 +/- 1072.2 | 3569.28 +/- 105.36 | 228.67 +/- 74.27 | 856.95 +/- 18.3 | 623.54 +/- 132.22 |
|  | L2026M | 33.41 +/- 27.26 | 1117.66 +/- 340.22 | 621.82 +/- 406.2 | 51.58 +/- 14.8 | 17.27 +/- 6.18 |
|  | S1986Y | 1.14 +/- 1.19 | 13.21 +/- 5.47 | 61.67 +/- 4.75 | 41.05 +/- 18.07 | 38.16 +/- 41.23 |

**Reagents and compounds used in the study**

| **Reagent** | **Manufacturer** | **Catalogue number or sequence (5’🡪 3’)** |
| --- | --- | --- |
| HiFi Cas9 Enzyme | IDT Technologies | 1081060 |
| Electroporation enhancer | IDT Technologies | 1075916 |
| SF 4D Nucleofector S Kit | Lonza | 197174 |
| CRISPR tracrRNA | IDT Technologies | 1072533 |
| G2032R_gRNA | IDT Technologies | ATCCTGGAACTGATGGAGGG |
| G2032R HDR Donor template | IDT Technologies | GTTGCCATCCGGGCTTTACGCAAATAAGTAAGAAGGTCTCGTCCTTCCATCAGTTCCAGGATAATGTATTGGGGTTCATTCAGCA |
| L2026M_gRNA | IDT Technologies | TGAACCCCAATACATTATCC |
| L2026M HDR Donor template | IDT Technologies | CGCAAATAAGTAAGAAGGTCTCCTCCCTCCATCAGTTCCATAATAATGTATTGGGGTTCATTCAGCAGACAAACTCCAAGCTG |
| S1986Y_gRNA | IDT Technologies | GAAGAAGGGTTCCACAGACC |
| S1986Y HDR Donor template | IDT Technologies | ATCAGATGTGCCTCCTTCAGGAATTCAATCTTCTCTTGGTCTGTGAAACCCTTCTTCAAAGTCTATACAACATAAAAACAAGTCAG |
| SuperScript^TM^ III First Strand Synthesis System | ThermoFisher Scientific | 18080051 |
| Mutant validation forward primer (Nested PCR 1) | IDT Technologies | CTTCCAAGGGATTGGGAGATTG |
| Mutant validation reverse primer (Nested PCR 1) | IDT Technologies | CCACTGTTGTTTGCTTCATCTC |
| Crizotinib | Selleck Chemicals | S1068 |
| Entectinib | TargetMol Chemicals | T3678 |
| Lorlatinib | Merck | PZ0039 |
| Ceritinib | TargetMol Chemicals | T1791 |
| Repotrectinib | Selleck Chemicals | S8583 |
| Cytotox Green | Sartorius | 4632 |
| Cultrex Type 2 | Bio-Techne | 3532-010-02 |
